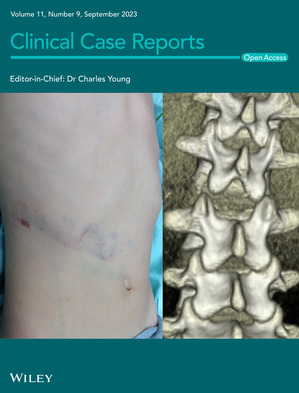# Cover Image

**DOI:** 10.1002/ccr3.8021

**Published:** 2023-10-09

**Authors:** Hirohito Hirata, Tadatsugu Morimoto, Masatsugu Tsukamoto, Takaomi Kobayashi, Tomohito Yoshihara, Yu Toda, Masaaki Mawatari

## Abstract

The cover image is based on the Case Report *Pediatric chance fracture with seatbelt syndrome: A case report* by Hirohito Hirata et al., https://doi.org/10.1002/ccr3.7886